# An evaluation of the Invisalign® Aligner Technique and consideration of the force system: a systematic review

**DOI:** 10.1186/s13643-023-02437-5

**Published:** 2024-01-27

**Authors:** Silvia Caruso, Maria Elena De Felice, Chiara Valenti, Stefano Pagano, Sara Caruso, Roberto Gatto, Guido Lombardo

**Affiliations:** 1 Department of Life, Health and Environmental Sciences, University of L’Aquila, 67100 L’Aquila, Italy; 2 CISAS “Giuseppe Colombo”, University of Padua, Via Venezia, 15, 35131 Padua, Italy; 3 Department of Medicine and Surgery, Faculty of Dentistry, University of Perugia, S. Andrea delle Fratte, 06156 Perugia, Italy

**Keywords:** Orthodontic appliances, Orthodontic treatment, Digital planning, Removable appliances

## Abstract

**Objective:**

Since its introduction 25 years ago, the Invisalign® system has undergone multiple digital and biomechanical evolutions and its effectiveness is often compared to traditional systems without considering the many differences which characterize them. The main aim of this systematic review is to look at the literature dealing with studies on teeth movements using the Invisalign® system and the management of these movements through digital planning and artificial intelligence.

**Materials and methods:**

The following electronic databases were searched: MEDLINE, Embase, the Cochrane Oral Health Group’s Trials Register, and CENTRAL. Unpublished studies were searched on ClinicalTrials.gov, the National Research Register, and Pro-Quest Dissertation Abstracts and Thesis database.

**Results:**

Twenty-four studies (15 retrospective, 5 prospective, 2 pilot, and 2 case–control) were included. The results of the analysis carried out on the available literature show that the Invisalign® system is recognized to be a valid alternative to conventional orthodontic treatment in no-extraction cases. The results are influenced by the methods for assessing the effectiveness of this technique and by the comparison bias of the traditional system with the innovative digital system.

**Conclusions:**

Since the introduction of SmartForce and SmartTrack material, the efficacy of the treatment has improved. There is still a shortage of high-quality evidence concerning the treatment modality. In order to make the treatment with the aligners more efficient, a correct management of the ClinCheck® software and a proper use of the biomechanics are necessary. The aligned force-driven system should be taken into account when developing the digital planning.

## Introduction

In 1997, two students from the University of Stanford revolutionized the way we have been practicing orthodontics by introducing the Invisalign® system which uses digital software to plan dental movements making them possible by using clear aligners which have replaced traditional brackets.

The aligners have proved to be an increasingly widespread solution for adults and growing patients who express the desire to resort to aesthetic and comfortable alternatives to the use of conventional fixed appliances. The adverse effects of traditional orthodontics, like periodontal diseases, are minimized by using a removable device which also allows patients to easily perform oral hygiene procedures [1, 2].

If compared to fixed orthodontic appliances, the greatest advantage of the clear aligner is the improvement of aesthetics and comfort for the patient. Furthermore, it is possible to control the force system of the tooth movements and manage them in a more accurate way thanks to the ClinCheck® software.

Notwithstanding the existence of a large body of literature related to the Invisalign® technology, a comprehensive study of its clinical performance has not yet been carried out and a synthesis of the evidence is lacking. Three systematic reviews on the accuracy and predictability of treatment with the Clear Aligners System have assessed the evidence related to the efficacy of clear aligner treatment (CAT) in controlling orthodontic tooth movement; however, these reviews date back to 2015, 2017, and 2018, respectively [3–5]. Further reviews have compared CAT with conventional brackets [6] and assessed the prediction of rotational tooth movements with aligners [7]. Because of the continuous improvement of the Invisalign® system and since reviews include studies that analyze different types of aligners, the findings should be interpreted with some caution. The two most notable innovations are the introduction of SmartForce features (2008), such as optimized attachments, pressure zones, and customized staging, and the SmartTrack aligner material (2011) which allows for a better range of force delivery and fit.

Therefore, the purpose of the present review is to re-evaluate the effectiveness of this treatment system by only considering research using the latest updates of the Invisalign® system.

Moreover, another aspect of this review is to introduce the concept that the aligned force-driven system should be taken into account when developing the digital planning. The teeth movements that occur are due to the combination of a pure mechanic movement together with a release of differential forces based on the extent and kind of correction.

## Materials and methods

### Ethics

Ethics research ethics committee (REC) approval was not required for this review.

### Registration and reporting

The systematic review followed the PRISMA (Preferred Reporting Items for Systematic Reviews and Meta-Analysis) guidelines.

### Search strategy

Detailed search strategies were developed and appropriately revised for each database, considering the differences in controlled vocabulary and syntax rules by the first author (S.C). The following electronic databases were searched: MEDLINE (via Ovid and PubMed, Appendix, from 1946 to August 28, 2017), Embase (via Ovid), the Cochrane Oral Health Group’s Trials Register, and CENTRAL. Unpublished studies were searched on ClinicalTrials.gov, the National Research Register, Pro-Quest Dissertation Abstracts, and Thesis database. The search attempted to identify all relevant studies irrespective of language. The reference lists of all eligible studies were examined for additional studies. A manual search was thoroughly performed to identify additional articles in the references of selected articles.

A systematic search in the medical literature, from inception to April 2023, was performed to identify all peer-reviewed articles potentially relevant to the review’s question. Our search strategy below was designed by an experienced information specialist.

((“Orthodontics”[MeSH Terms] OR “Orthodontic Appliances”[MeSH Terms] OR “Orthodontic Appliances, Removable”[MeSH Terms] OR “Orthodont*”[All Fields] OR “Orthodontics, Corrective”[MeSH Terms] OR “Orthodontics, Preventive”[MeSH Terms] OR “Orthodontics, Interceptive”[MeSH Terms] OR “Orthodontic Appliances, Removable”[All Fields] OR “Orthodontics”[All Fields] OR “Orthodontic Appliances”[All Fields] OR “Malocclusion*”[All Fields] OR “ Malocclusion”[MeSH Terms] OR “Invisalign treatment”[All Fields] OR “Invisalign”[All Fields] OR “Invisalign”[title/abstract] OR “Digital Treatment Planning”[All Fields] OR “ClinCheck Software”[All Fields] OR “iTero”[All Fields] OR “Clear Aligners”[All Fields] OR “Aligners”[All Fields] AND “Treatment Outcome”[All Fields]) NOT (Systematic Review [Publication Type] OR Review [Publication Type] OR Meta-Analysis [Publication Type] OR Comment [Publication Type] OR Congress [Publication Type] OR Editorial [Publication Type] OR Case Reports [Publication Type] OR Clinical Conference [Publication Type] OR Comment [Publication Type] OR Consensus Development Conference [Publication Type]).

### Types of studies

Articles were included if they evaluated the predictability of teeth movement with clear aligners or if aligner treatment outcome was compared to fixed appliance therapy. Randomized clinical trials (RCTs), controlled clinical trials (CCTs), and prospective and retrospective studies were considered eligible for inclusion in this review. Case reports, in vitro studies, author’s letters, and studies with surgical interventions were excluded.

### PICOs

The review was conducted based on the Population, Inclusion, Comparison, Outcome (PICOs) format: “Is the Invisalign® System effective in performing orthodontic movements if compared with fixed orthodontic appliance or with teeth movement planned on the ClinCheck® software? (Table 1).

**Table 1:**
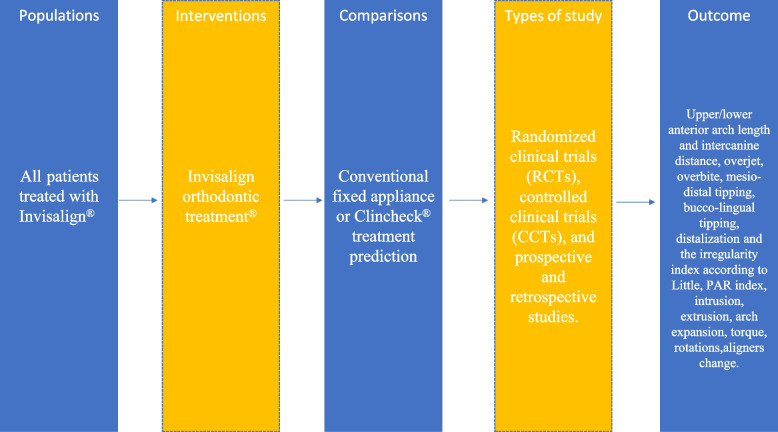
A diagram to illustrate the study population, interventions and comparisons, types of study, and patient-relevant outcomes

### Population

Orthodontic adult patients (≥ 18 years of age) who were treated with Invisalign® either as the intervention or as the control group.

### Intervention and comparators

The Invisalign® treatment was compared both to fixed orthodontic appliances and to predicted tooth movement based on ClinCheck® Software. All other aligner systems have been excluded.

### Outcome

Any result on clinical efficiency, effectiveness, treatment outcomes, movement accuracy, or predicted tooth movement in ClinCheck® software of Invisalign® treatment, including changes in alignment or occlusion, treatment duration, and comparison with fixed appliance.

Evaluated parameters were upper/lower anterior arch length and intercanine distance, overjet, overbite, mesio-distal tipping, bucco-lingual tipping, distalization, and the irregularity index according to Little [8].

### Selection of studies

**Fig. 1:**
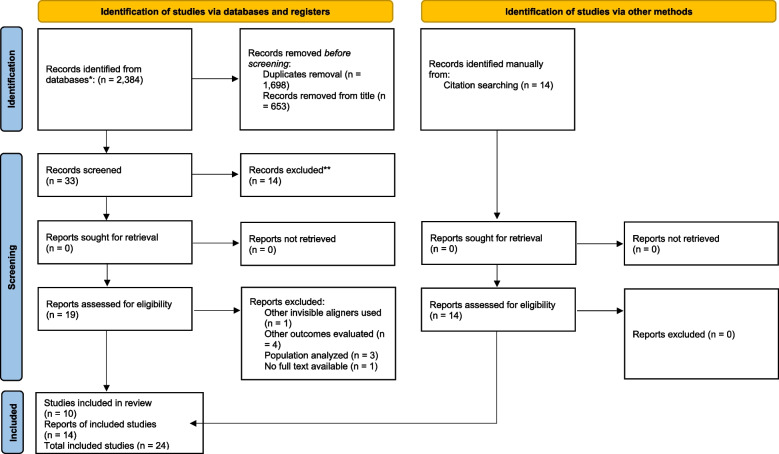
Flow diagram for the selection of studies according to PRISMA (diagram from: Moher D, Liberati A, Tetzlaff J, Altman DG, The PRISMA Group (2009). Preferred Reporting Items for Systematic Reviews and Meta-Analyses: The PRISMA Statement. PLoS Med 6(7): e1000097

Study selection was performed independently and in duplicate by two authors of the review, who were not blinded to the identity of the authors of the studies, their institutions, and the results of their research. The study selection procedure included title-reading, abstract-reading, and full-text-reading stages. The calculated coefficient of agreement between the two reviewers who screened the title and abstract of the retrieved records indicated high agreement (*k* value = 0.87). After the exclusion of non-eligible studies, the full report of publications considered eligible for inclusion by either author was obtained and assessed independently (Fig. 1).

### Data extraction and management

The first two authors performed data extraction independently and in duplicate. Disagreements were resolved by discussion with the involvement of two collaborators (the third author and the last author). Data collection forms were used to record the desired information. The following data were collected on a customized data collection form:

- Author/title/year of study
- Design/setting of the study
- Number/age
- Intervention and comparator/treatment duration
- Type of clinical outcome
- Method of outcome assessment

### Quality assessment

The quality of the included studies was assessed using the Newcastle–Ottawa Scale (NOS), an assessment scale for assessing the quality of non-randomized studies [9].

### Dealing with missing data

We contacted study authors via e-mail to request missing data where necessary. In case of no response or no provision of the missing data, only the available reported data were analyzed.

## Results

Twenty-four studies (15 retrospective studies, 5 prospective, 2 pilot studies, and 2 case–control) were included. Respecting the selection, comparability, and outcome criteria, four of the twenty-four analyzed studies [10–17] were awarded the maximum number of points 9/9. The lack of standardized outcome reporting, and the high amount of clinical and methodological heterogeneity across the included studies precluded the conduct of a meta-analysis in achieving pooled estimates of effects. The results from the included studies were thus reported narratively. However, there was substantial consistency among studies that the Invisalign® system is a viable alternative to conventional orthodontic therapy in the correction of mild to moderate malocclusions in non-growing patients that do not require extraction.

Moreover, Invisalign® aligners can predictably level, tip, and derotate teeth (except for cuspids and premolars). On the other hand, limited efficacy was identified in arch expansion through bodily tooth movement, corrections of occlusal contacts, and larger antero-posterior and vertical discrepancies. The sample size in individual studies ranged from 20 to 200, with a total of 1391 patients. Age at the start of the aligner’s treatment in the evaluated samples ranged from 13 to 75 years (Table 2).

**Table 2:** Characteristics of included studies

Author, year	Study design	Population	Intervention	Comparison	Outcomes
Morales-Burruezo et al., 2020 [18]	Retrospective study	114 participants; aged 18–75 years	-Efficacy for arch expansion (transverse distance variation) at the level of upper maxillary canine, first and second premolars, and first and second molars -Efficacy for upper maxillary first molars rotation and inclination -Predictability of ClinCheck® software movements’ previsions	ClinCheck® software (planned vs achieved)	**Transverse expansion** -Canines (1.87 SD 1.78 mm, + 6.31%) -First premolars (3.14 SD 2.25 mm, + 8.73%) -Second premolars (3.45 SD 2.09 mm, + 8.42%) -First molar (2.57 SD 1.83 mm, + 5.64%) -Second molar (0.45 SD 1.83 mm, + 0.54%) **Inclination right first molar**: 2.26 SD 4.76 mm **Inclination left first molar**: 2.13 SD 4.09 mm **Rotation right first molar**: 2.22 SD 4.37 mm **Rotation left first molar**: 2.46 ± 3.75 mm **Predictability** -Intercanine distance: 0.63 SD 0.75 (74.8%) -First premolar: 0.77 SD 1.44 mm (80.3%) -Second premolar: 0.81 SD 1.26 mm (81.0%) -First molar: 0.69 SD 1.21 mm (79.1%) -Second molar: 0.25 SD 1.97 mm (65.2%) -Inclination right first molar: − 0.42 SD 3.36 mm (123.5%) -Inclination left first molar: − 0.88 SD 2.73 mm (170.4%) -Rotation right first molar: 0.54 SD 3.05 mm (80.4%) Rotation left first molar: − 0.34 SD 3.57 mm (115.3%)
Houle et al., 2017 [19]	Retrospective study	64 participants Aged: 18–61 years (mean age 31.2 years)	Accuracy of transverse width measured at: - Level of canine tip and gingival margin -First premolar tip and gingival margin -Second premolar tip and gingival margin -First molar tip and gingival margin	ClinCheck® software (planned vs achieved)	**Predictability of transverse expansion: ** **Upper arch**-Canine tip: 0.22 SD 0.74 mm (88.7%) -Canine gingival margin: 0.6 SD 1.02 mm (67.8%) -First premolar tip: 0.58 SD 1.14 mm (84.7%) -First premolar gingival margin: 1.09 SD 1.22 mm (67.6%) -Second premolar tip: 0.75 SD 1.54 mm (81.7%) -Second premolar gingival margin: 1.3 SD 1.61 mm (62.3%) -First molar tip: 0.77 SD 1.84 mm (76.6%) -First molar gingival margin: 1.43 SD 1.9 mm (52.9%) **Lower arch** -Canine tip: − 0.08 SD 0.81 mm (100%) -Canine gingival margin: 0.65 SD 1.01 mm (61%) -First premolar tip: 0.07 SD 0.96 mm (96.9%) -First premolar gingival margin: 0.27 SD 1.00 mm (88.4%) -Second premolar tip: 0.07 SD 1.15 mm (98.9%) -Second premolar gingival margin: 0.38 SD 1.16 mm (85.5%) -First molar tip: 0.03 SD 1.33 mm (100%) -First molar gingival margin: 0.54 SD 1.34 mm (70.7%)
Krieger et al., 2012 [20]	Extended study based on previous pilot study	50 participants; Aged 15–63 years (mean 33 SD 11.9)	Accuracy of: -Upper/lower anterior arch length -Intercanine distance -Overjet -Overbite -Dental midline shift -Irregularity index according to little	ClinCheck® software (planned vs achieved)	**Little’s irregularity index:** -Upper dentition: from 5.39 SD 2.23 mm before treatment to 1.57 SD 0.98 mm post-treatment -Lower dentition: from 5.96 SD 2.39 mm to 0.82 SD 0.50 mm -Difference between clinically achieved and planned reduction of Little’s irregularity index was 0.04 SD 0.65 mm for the upper anterior arch and 0.01 SD 0.48 mm for the lower anterior arch **Upper inter-canine distance** From 33.51 SD 2.05 mm pre-treatment to 33.67 SD 2.00 mm post-treatment -Difference between clinically achieved and planned upper intercanine distance variation was − 0.13 SD − 0.59 **Lower inter-canine distance**: From 24.57 SD 1.69 mm to 25.27 SD 1.52 mm **Overjet** From 4.31 SD 1.43 mm to 2.94 SD 0.94 mm -Overjet variation − 0.34 SD 0.54 mm **Overbite** From 4.05 SD 1.50 mm to 3.49 SD 1.19 mm -Overbite variation − 0.71 SD 0.87 mm **Dental midline shift** From 1.38 SD 0.99 mm to 0.99 SD 0.89 mm -Dental midline shift − 0.24 SD 0.46 mm
Lanteri et al. 2018 [14]	Retrospective study	200 participants Aged 14–56 years	-Anterior dental crowding measured with little irregularity index or Peer Assessment Rating index (PAR)	Smart track aligners vs conventional fixed appliances	**Invisalign** **-**63/100 (80.9%) fully resolved their anterior dental crowding and did not need any refinement - PAR index: 22.5 SD 7 to 3.5 SD 3 - Maxillary Little Index Pre-treatment 23% moderate/62% minimal Post-treatment 100% perfect alignment -Mandibular Little Index Pre-treatment 12% severe/36% moderate/52% minimal Post-treatment 92% perfect alignment/8% minimal Treatment duration 14 SD 7 months **Conventional fixed appliance** - PAR index: 24.0 SD 6 to 4.5 SD 4 - Maxillary Little Index Pre-treatment 31% moderate/69% minimal Post-treatment 100% perfect alignment -Mandibular Little Index Pre-treatment 16% severe/32% moderate/52% minimal Post-treatment 88% perfect alignment/12% minimal Treatment duration 19 SD 4 months
Simon et al., 2014 [11]	Case–control (split mouth)	30 participants Aged 13–72 years	Accuracy: -Upper incisor torque > 10° -Premolar derotation > 10° -Upper molar distalization > 1.5 mm	Invisalign® with and without auxiliaries (attachments and staging)	**Accuracy** **Upper Incisor Torque > 10°** Invisalign with horizontal ellipsoid attachments vs Invisalign with power bridges: 51.5% SD 0.2 vs. 49.1%SD 0.2 **Premolar derotation > 10°** Invisalign with optimized rotation attachment vs Invisalign without auxiliaries: 37.5% SD 0.3 vs. 42.4% SD 0.3 **Upper molar distalization > 1.5 mm** Invisalign with horizontal beveled gingival attachment vs Invisalign without auxiliaries: 88.4% SD 0.3 vs. 86.9% SD 0.16 **The overall accuracy** Upper incisor torque > 10°: 42% Premolar derotation > 10°: 40% Upper molar distalization > 1.5 mm: 87%
Zhou et al. 2020 [21]	Retrospective study	20 participants Aged 20–45 years (mean 28.5 SD 6.3)	Accuracy of transverse width measured at: - Level of canine tip -First premolar tip -Second premolar tip -First molar tip Maxillary basal bone width variations Maxillary alveolar bone (buccal and palatal ridge crest) width variation Difference of maxillary first molar tipping	ClinCheck® software (planned vs achieved)	**Transverse expansion** -Canine 1.44 SD 0.60 mm -First premolar 1.74 SD 0.84 mm -Second premolar 1.57 SD 0.96 mm **Predictability of transverse expansion** -Canine tip: 0.33 SD 0.26 mm (79.75%) -First premolar tips: 0.53 SD 0.45 mm (76.1%) -Second premolar gingival: 0.65 SD 0.76 (73.3%) -First molar tip 0.74 SD 0.73 (68.3%) **Difference in the basal bone width** 0.04 SD 0.18 mm **Maxillary alveolar bone arch width** - Buccal ridge crest: 0.87 SD 0.63 mm - Palatal ridge crest 0.75 SD 0.80 mm **Maxillary first molar tipping** 2.07 SD 3.3
Solano-Mendoza et al., 2017 [22]	Retrospective study	116 participants Mean age 36.6 (SD 11.5)	Accuracy of transverse width measured at: - level of canine tip and gingival margin -first premolar tip and gingival margin -second premolar tip and gingival margin -first molar tip and gingival margin	ClinCheck® software (planned vs achieved)	**Predictability** -Canine tip: 94.2% - Canine gingival margin: 76.5% -First premolar tip: 89.7% -First premolar gingival margin: 84.2% -Second premolar tip: 92.1% -Second premolar gingival margin: 88.9% -First molar tip: 88.6% -First molar gingival margin: 87.7%
Riede et al., 2021 [23]	Retrospective study	30 participants Aged 13–50 years	Accuracy of expansion	ClinCheck® software (planned vs achieved)	**Transverse expansion** -Canine tip: 0.4 SD 0.3 mm -Canine gingival margin: 0.45 SD 0.3 mm -First premolar tip: 0.5 SD 0.25 mm -First premolar gingival margin: 0.4 SD 0.2 mm -Second premolar tip: 0.5 SD 0.3 mm -Second premolar gingival margin: 0.5 SD 0.45 mm -First molar tip: 0.5 SD 0.35 mm -First molar gingival margin: 0.5 SD 0.3 mm -Distobuccal cusp tip in first and second molars 2.9 SD 1.9° and 2.9 SD 2.4°, respectively Participants achieving the width variation planned by ClinCheck software for each site of the following upper maxillary teeth: -Canine cusp (46.6%) -Canine gingival margin (28.3%) -First premolar cusp (41.7%) - First premolar gingival margin (46.7%) -Second premolar cusp (50%) -Second premolar gingival margin (56.7%1) - First molar cusp (40%) -First molar gingival margin (50%)
Gu et al., 2017 [16]	Case–control	96 participants Mean age 22.1 SD 7.9 and 26 SD 9.7	-Peer Assessment Rating (PAR) Index -Treatment duration	Invisalign® vs conventional fixed appliances	-Both intervention and control groups achieved a statistically significant clinical improvement of PAR index (> 30% of score reduction) -Fixed orthodontic appliance was better than Invisalign in resolving malocclusion based on PAR index scores -Fixed orthodontic appliance was more effective than Invisalign in reducing the mean percentage of PAR index -Invisalign treatment was faster than fixed orthodontic appliance: (13.35 vs 19.08 months)
Grünheid et al., 2017 [24]	Retrospective study	30 participants Age 21.6 SD 9.8	Accuracy of: -Mesial-distal -Facial-lingual - Occlusal-gingival -Tip -Torque -Rotation	ClinCheck® software (planned vs achieved)	**Accuracy** ***Mesial-distal*** Maxilla -Central incisor: − 0.06 SD 0.4 mm -Lateral incisor: − 0.14 SD 0.39 mm -Canine: − 0.11 SD 0.51 mm -First premolar: 0.02 SD 0.47 mm -Second premolar: 0.19 SD 0.65 mm -First molar: 0.27 SD 0.30 mm -Second molar: 0.07 SD 0.81 Mandible -Central incisor: 0.12 SD 0.44 mm -Lateral incisor: − 0.8 SD 0.62 -Canine: − 0.11 SD 0.72 mm -First premolar: 0.02 SD 0.44 mm -Second premolar: 0.13 SD 0.57 mm -First molar 0.12 SD 0.34 mm -Second molar 0.02 SD 0.50 mm ***Facial-lingual*** Maxilla -Central incisor: − 0.45 SD 0.64 mm -Lateral incisor: 0.01 SD 0.66 mm -Canine: 0.11 SD 0.60 mm -First premolar: 0.15 SD 0.53 mm -Second premolar: 0.20 SD 0.63 mm -First molar: 0.23 SD 0.62 mm -Second molar: 0.30 SD 0.79 Mandible -Central incisor: 0.11 SD 0.56 mm -Lateral incisor: − 0.01 SD 0.51 mm -Canine: − 0.26 SD 0.49 mm -First premolar: 0.05 SD 0.62 mm -Second premolar: 0.09 SD 0.59 mm -First molar: − 0.08 SD 0.52 mm -Second molar − 0.017 SD 0.39 mm ***Occlusal-gingival*** Maxilla -Central incisor: − 0.30 SD 0.28 mm -Lateral incisor: − 0.03 SD 0.26 mm -Canine: − 0.02 SD 0.24 mm -First premolar: 0.06 SD 0.19 mm -Second premolar: 0.01 SD 0.22 mm -First molar: − 0.02 SD 0.14 mm -Second molar: − 0.13 SD 0.29 mm Mandible -Central incisor: − 0.14 SD 0.21 mm -Lateral incisor: − 0.10 SD 0.22 mm -Canine: − 0.01 SD 0.21 mm -First premolar: 0.09 SD 0.24 mm -Second premolar: 0.04 SD 0.21 mm -First molar: − 0.01 SD 0.15 mm -Second molar: 0.047 SD 0.16 mm ***Tip*** Maxilla -Central incisor: − 0.42 SD 1.57° -Lateral incisor: 0.35 SD 2.36° -Canine: 0.31 SD 2.24° -First premolar: − 0.18 SD 1.96° -Second premolar: − 0.82 SD 3.63° -First molar: − 1.06 SD 1.4° -Second molar: 0.41 SD 5.18° Mandible -Central incisor: − 0.36 SD 1.81° -Lateral incisor: 0.51 SD 2.75° -Canine: 0.39 SD 3.11° -First premolar: 0.16 SD 2.04° -Second premolar: − 0.55 SD 2.55° -First molar: − 0.38 SD 1.35° -Second molar: 1.07 SD 3.06° ***Torque*** Maxilla -Central incisor: 1.75 SD 2.86° -Lateral incisor 0.08 SD 2.93° -Canine: − 0.048 SD 2.55° -First premolar: − 0.74 SD 2.40° -Second premolar: − 1.18 SD 3.27° -First molar: − 1.45 SD 2.37° -Second molar: − 2.13 SD 4.19° Mandible -Central incisor: − 0.66 SD 2.61° -Lateral incisor: − 0.29 SD 2.34° -Canine: − 1.60 SD 2.04° -First premolar: − 0.60 SD 2.53° -Second premolar: − 0.74 SD 3.05° -First molar: − 0.85 SD 2.41° -Second molar: − 1.09 SD 2.13° ***Rotation*** Maxilla -Central incisor: − 0.33 SD 2.80° -Lateral incisor: 0.70 SD 3.23° -Canine: 0.19 SD 2.31° -First premolar: − 0.48 SD 1.48° -Second premolar: − 0.70 SD 1.95° -First molar: − 0.52 SD 1.58° -Second molar: 0.06 SD 2.20° Mandible -Central incisor: − 0.60 SD 1.71° -Lateral incisor: − 0.99 SD 2.28° -Canine: 0.88 SD 3.14° -First premolar: − 1.71 SD 2.91° -Second premolar: − 0.88 SD 3.86° -First molar: − 0.30 SD 1.07° -Second molar 0.29 SD 2.66° *Statistically significant discrepancy with ClinCheck® prevision involves* -Upper central incisors (facial-lingual and occlusal-gingival movements) -Upper second premolar and upper first molar (mesial-distal and facial-lingual movements) -Upper second molar (occlusal-gingival movements) -Lower central and lateral incisors (occlusal-gingival movements) -Upper central incisor (torque) -Upper first molar (tip and torque) -Second lower premolar and molar (torque) -Lower lateral incisor (rotation) -Lower canine (torque and rotation) -First and second lower premolars (rotation) -Lower second molar (tip)
Houili et al., 2020 [12]	Prospective clinical study	38 participants Mean age 36 years	Accuracy -Mesial-distal crown tip -Buccal-lingual crown tip -Extrusion -Intrusion -Mesial-distal rotation	ClinCheck® software (planned vs achieved)	**Accuracy** The mean accuracy of Invisalign for all tooth movements was 50% -Rotation (46%) -Buccal-lingual crown tip (56%) -Mesial rotation of the mandibular first molar (28%) -Intrusion of the maxillary central incisor (33%) -Intrusion of the mandibular incisors (35%) -Buccal crown tip of the maxillary second molar (35%) -Distal rotation of the maxillary canine (37%) -Extrusion of the mandibular second molar (37%) -Distal crown tip of the mandibular second molar (50%) -Intrusion of the mandibular second molar (51%) -Mesial rotation of the maxillary canine (52%) -Extrusion of the maxillary central incisor (56%) -The lingual crown tip of the maxillary second molar (61%) - Buccal crown tip of the maxillary second premolar (61%) -Distal crown tip of the maxillary second molar (63%) -Labial crown tip of the maxillary lateral incisor (70%) -Buccal crown tip of the mandibular second premolar (70%)
Kassas et al., 2013 [17]	Retrospective study	31 participants Mean age 35.2 ± 13.2 years	Model Grading System (MGS) of the American Board of Orthodontics: -Alignment -Marginal ridges -Buccolingual inclination -Occlusal contacts -Occlusal relations -Overjet -Interproximal contacts		The mean scores of all of the MGS categories were improved after treatment, with the exceptions of the occlusal contacts and occlusal relationships categories The improvements were statistically significant in scores: -Alignment category: 15.16 SD 5.00 vs. 6.00 SD 3.78 -Buccolingual inclination category: 7.00 SD 3.14 vs. 6.26 SD 3.58 -Total MGS score: 45.03 SD 7.47 vs. 35.87 SD 9.36 *Using the ABO criteria* -1 case (3%) received a passing score -22 cases (71%) failed -8 cases (26%) were considered borderline
Pavoni et al., 2011 [25]		40 participants Mean age Self-ligating 15 years Mean age Invisalign® Group 18 years	Transversal expansion: -Intercanine width (lingual) -Intercanine width (cusp) -First interpremolar width (lingual) -First interpremolar width (fossa) -Second interpremolar width (lingual) -Second interpremolar width (fossa) -Intermolar width (lingual) -Intermolar width (fossa) -Arch depth -Arch perimeter	Self-ligating vs Invisalign®	**Self-ligating group** -Intercanine width (cusp) showed a significant increase from T1 to T2: 3.15 mm -First interpremolar widths (lingual and cusp) had significant increases of 3.40 mm and 2.45 mm, respectively -Second interpremolar widths (lingual and cusp), with significant increases of 2.50 mm and 2.15 mm, respectively **Invisalign® group** -Second interpremolar width at the fossa point (0.45 mm) -Intermolar widths at the fossa (0.50 mm) Significant difference was found between the 2 groups for the intercanine widths, the change at the cusp was significantly larger in the self-ligating group (2.65 mm) The comparison between the two groups of the first interpremolar measurements showed an improvement in the self-ligating subjects significantly bigger at the lingual point (2.30 mm), and at the cusp (3.35 mm), similar to the second interpremolar widths (lingual and cusp), with a significant increase of 1.85 mm and 2.05 mm, respectively
Drake et al., 2012 [26]	Prospective single-center clinical trial	15 new participants (weekly aligner group)	-ΔU1(x) refers to the distance between lines drawn through the midpoint of the incisal edges of the superimposed target tooth perpendicular to the A-P axis (the plane of prescribed tooth movement) - ΔU1(s) is the length of the line connecting the midpoint of the incisal edges of the superimposed target tooth - ΔApex refers to the length of a line connecting the change in apex of the superimposed target tooth - Rotation angle is the angle created by the intersection of lines drawn from the midpoint of the incisal edge to the apex of the target tooth. The apex of this angle is considered the center of rotation - Tooth length refers to the distance from the midpoint of the incisal edge to the apex of the target tooth from the initial X-ray - Crown length is the portion of the tooth length that is coronal to the bone - Bone to C-rot. is the section of tooth length between the center of rotation and a line connecting the most coronal aspect of the faciolingual crestal bone - ΔU1(o) refers to the A-P change in the midpoint of the superimposed incisal edge of the opposite central incisor, the one that was not the target tooth - ΔU1(t) refers to the distance between the midpoint of the superimposed incisal edge of the contralateral central incisor, to the midpoint of the incisal edge of the target tooth	37 participants previously collected (biweekly aligner control group)	No overall difference in OTM (orthodontic tooth movement) was detected between the groups, with mean total OTM of 1.11 mm SD 0.30 and 1.07 mm SD 0.33 for the weekly aligner and biweekly control groups, respectively Also, no difference was detected in the weekly OTM of the weekly aligner versus biweekly control groups overall (*P* = 0.812) or between any 2-week prescription cycle for the weekly aligner and biweekly control groups However, 4.4 times more OTM occurred during the first week than the second week of aligner wear (*P* < 0.001) for the combined groups, considering all 2-week periods
Ravera et al., 2016 [15]	Multicenter retrospective study	20 participants Mean age 29.73 years	Bodily maxillary molar distalization	ClinCheck® software (planned vs achieved)	**Bodily distalization** -Upper first molar**:** 2.25 mm -Upper second molar: 2.52 mm
Duncan et al., 2016 [27]	A retrospective chart review	61 participants	Arch Expansion Interproximal reduction Lowe incisor position and angulation	ClinCheck® software (planned vs achieved)	**Differences in mean (T0-T1)** **Mild crowding** OVJ**:** 0.73 mm **Moderate crowding** OVJ: 0.73 mm OVB: 0.68 mm **Severe crowding** OVJ:1.32 mm L1-NB: − 4.70° L1-NB: − 1.55 mm L1-MPA: − 3.94° L1-APog: − 4.82° L1-APog: − 1.74 mm
Grunheid et al., 2016 [28]	Retrospective cohort study	60 participants Mean age 25/26 years	-Buccolingual inclination of the mandibular canines -Intercanine distance	Invisalign® vs conventional fixed appliances	**Difference (T2-T1)** **Clear aligner** Inclination: 0.7 SD 2.5° Distance: 0.7 SD 1.5 mm **Fixed appliance** Inclination: − 1.9 SD 5.1° Distance: − 0.1 SD 2.4 mm
Khosravi et al., 2017 [13]	Retrospective study	120 participants: -68 with a normal overbite -40 with deepbite -12 with openbite Mean age 18 years or older	Overbite changes	No control group	**Normal overbite** -Proclination of maxillary incisors (U1-NA): 0.7° and (L1-NB) 0.6° - Anterior facial height: + 0.7 mm -Mandibular plane angle: + 0.4° **Deep bite** -1.5-mm median opening of the overbite -Proclination of the mandibular incisors and intrusion of the maxillary incisors -Extrusion of mandibular first and second molars: 0.5 mm on average -Proclination of the mandibular incisors was the main mechanism of bite opening **Open bite** -A median deepening of 1.5 mm -Extrusion of the maxillary and mandibular incisors: (U1-PP) 0.9 mm and (L1-MP) 0.8 mm
Chisari et al., 2014 [29]	Prospective single-center clinical trial	30 participants Ages 19 to 64 years old	Assessment of the impacts of age, sex, root length, bone levels, and bone quality on orthodontic tooth movement	No control group	-The rate of movement decreases from ages 18 to 35 years -A slightly increasing rate from ages 35 to 50 and a decreasing rate from ages 50 to 70 -The correlation was significant between the percentage of the goal achieved and the cone-beam computed tomography superimposed linear measures of tooth movement -A significant negative correlation was found between tooth movement and the measurement apex to the center of rotation, but bone quality, as measured by fractal dimension, was not correlated with movement
Hennessy et al., 2016 [30]	Prospective clinical trial	44 participants Mean age 26.5 years SD 7.7	Mandibular incisor proclination	Invisalign® vs conventional fixed appliances	**Mandibular incisor proclination** -Fixed appliances: 5.3° SD 4.3° -Clear aligners: 3.4° SD 3.2°
Charalampakis et al., 2018 [10]	Retrospective study	20 subjects Mean age 37 years	-Horizontal displacements - Vertical displacements - Intercanine and interpremolar widths -Mesiodistal rotations	ClinCheck® software (planned vs achieved)	**Horizontal displacements** *Median difference (predicted-achieved)* -Maxillary central incisors horizontal (mm): 0.25 -Maxillary canines horizontal (mm): 0.20 **Vertical displacements** *Median difference (predicted-achieved)* -Maxillary central incisors intrusion (mm): 1.50 -Maxillary lateral incisors intrusion (mm): 1.10 - Mandibular incisors intrusion (mm): 0.80 -Mandibular canines vertical (mm): 0.30 - **Intercanine and interpremolar widths** *Median difference (predicted-achieved)* -Maxillary intercanine width (mm): 0.45 **Mesiodistal rotations** *Median difference (predicted-achieved)* -Maxillary central incisors rotation (°): 2 - Maxillary lateral incisors rotation (°): 1.85 - Maxillary canines rotation (°): 3.05 - Maxillary premolars rotation (°): 0.90 - Mandibular incisors rotation (°): 1.85 - Mandibular canines rotation (°): 2.45 - Mandibular premolars rotation (°): 1.90
Buschang et al., 2015 [31]	Prospective clinical study	27 participants No age indication	OGS scores: -Alignment -Marginal ridges -Buccolingual inclination -Occlusal contacts -Occlusal relations -Overjet -Interproximal contacts	ClinCheck® software (planned vs achieved)	-Differences were greatest for alignment, marginal ridges, and occlusal contacts -Differences for occlusal relations were also highly significant
Dai et al., 2019 [32]	Retrospective study	30 participants Mean age 19.4 ± 6.3 years	First premolar extractions with Invisalign: Achieved vs predicted movements of maxillary first molars and central incisors	ClinCheck® software (planned vs achieved)	**Difference (predicted and achieved)** **Central incisors** U1_Torque: − 5.16 SD 5.92° U1_LLT: 2.12 SD 1.51 mm U1_OGT: − 0.50 SD 1.17 mm **Maxillary first molars** U6_Angulation: 5.86 SD 3.51° U6MC_MDT: 2.26 SD 1.58 mm U6DC_ MDT: 2.31 SD 1.67 mm U6MC_OGT: 0.61 SD 0.89 mm U6DC_OGT: 0.01 SD 0.91 mm
Sfrondrini et al., 2018 [33]	Retrospective study	75 participants: -25 aligners -25 conventional fixed appliance -25 self-ligating appliance	Control of upper incisor torque: -11^SnaSnp -11^Ocl -I + TVL	Invisalign® vs -Conventional fixed appliance - Self-ligating appliance	**11^SnaSnp** Conventional: 6.11° SD 3.91 Self-ligating: 5.64° SD 3.27 Aligner: 5.13° SD 3.23 **11^Ocl** Conventional: 6.88° SD 4.28 Self-ligating: 5.17° SD 3.10 Aligner: 4.60° SD 3.46 **I + TVL** Conventional: 1.56 mm SD 0.47 Self-ligating: 1.62 mm SD 0.66 Aligner: 1.47 mm SD 0.57

### Qualitative synthesis of the included studies

Five studies [18, 22, 24, 26–28, 31, 33] reported an 8/9 points as they received ½ in the comparability criteria. Two studies [21, 23, 30] reported 7/9 points as they received one point in the comparability criteria and 2/3 points in the outcome criteria. Finally, three studies [11, 19, 20, 25, 29] received 6/9 points of which 2 missing in the selection criteria and one missing in the comparability criteria. All of them exceeded 5 points, and thus, they are of high quality as seen in the table (Tables 2 and 3).

**Table 3:** Newcastle—Ottawa Quality Assessment Scale (NOS) for case control and cohort studies

Study	Selection (max 1 star for each of the 4 items)				Comparability (max 2 stars for the 1 item)	Outcome (max 1 star for each of the 3 items)			Total stars
Buschang 2015 [31]	*	*	*	*	*	*	*	*	8/9
Charalampakis 2018 [10]	*	*	*	*	**	*	*	*	9/9
Chisari 2014 [29]	*	*			*	*	*	*	6/9
Dai 2019 [32]	*	*	*	*	**	*	*	*	9/9
Drake 2012 [26]	*	*	*	*	*	*	*	*	8/9
Duncan 2016 [27]	*	*	*	*	*	*	*	*	8/9
Grünheid 2016 [28]	*	*	*	*	*	*	*	*	8/9
Grünheid 2017 [24]	*	*	*	*	*	*	*	*	8/9
Gu 2017 [16]	*	*	*	*	**	*	*	*	9/9
Haouili 2020 [12]	*	*	*	*	**	*	*	*	9/9
Hennessy 2016 [30]	*	*	*	*	*	*	*		7/9
Houle 2017 [28]	*	*			*	*	*	*	6/9
Kassas 2013 [17]	*	*	*	*	**	*	*	*	9/9
Khosravi 2017 [13]	*	*	*	*	**	*	*	*	9/9
Krieger 2012 [20]	*	*			*	*	*	*	6/9
Lanteri 2018 [14]	*	*	*	*	**	*	*	*	9/9
Morales-Burruezo 2020 [18]	*	*	*	*	*	*	*	*	8/9
Pavoni 2011 [25]	*	*			*	*	*	*	6/9
Ravera 2016 [15]	*	*	*	*	**	*	*	*	9/9
Riede 2021 [23]	*	*	*	*	*	*	*		7/9
Sfondrini 2018 [33]	*	*	*	*	*	*	*	*	8/9
Simon 2014 [11]	*	*			*	*	*	*	6/9
Solano-Mendoza 2016 [22]	*	*	*	*	*	*	*	*	8/9
Zhou 2020 [21]	*	*	*	*	*	*	*		7/9

### Clinical findings

#### Transverse changes

Six studies have focused attention on the efficacy and predictability of the transversal expansion with Invisalign®.

##### Efficacy

In 2011, Pavoni et al. showed that the Invisalign® group, made of 20 participants, had a statistically significant increase in transverse dimension: second interpremolar width at the fossa point (0.45 mm), intermolar widths at the fossa (0.50 mm), and canine cusp width (0.50 mm). Nevertheless, these values are lower than the ones from the self-ligating group (20 patients), exactly 2.50 mm for the fossa point, and 0.90 mm for intermolar widths. Moreover, a significant difference was found between the two groups for the intercanine widths, and the change at the cusp was significantly larger in the self-ligating group (2.65 mm) [26].

A study with sixty-one patients of Duncan et al. stated that the arch width increased more in patients at the initial moderate and severe crowding. The mean increase in intermolar width was 1.65 mm in the mild crowding group, 1.86 mm in the moderate group, and 2.65 mm in the severe group. Interpremolar widths increased 1.57 mm, 2.52 mm, and 3.19 mm, respectively, and intercanine widths increased 1.28 mm, 1.77 mm, and 1.74 mm, respectively. The results revealed that buccal arch expansion played a significant role in crowding management [27].

Grunheid et al. assessed the buccolingual inclination of mandibular canines and their intercanine distance in sixty patients treated with clear aligner (30) and fixed appliance (30). The buccolingual inclination was greater in the aligner group than in the fixed appliance group at T2 but the canines appears more upright in the fixed appliance group [28].

In 2016, the average expansion obtained in 116 patients was 1.38 mm at cusp canine width, 0.54 mm at canine gingival width, 1.39 mm at first premolar gingival width, 1.25 mm at second premolar gingival width, and 0.56 mm at molar gingival width. Despite that, the expansion planned by the final ClinCheck® software is not predictable at canine cusp and gingival width, first premolar cusp and gingival width, second premolar cusp and gingival width, molar cusp and gingival width, and canine depth [22].

Houle et al. found a mean difference between planned and achieved teeth movements, exactl 0.22 mm for the canine crown, 0.6 mm for the canine gingival point, 0.58 mm for the first premolar crown, 1.09 mm for the first premolar gingival point, 0.75 mm for the second premolar crown, 1.3 mm for the second premolar gingival point, 0.77 for the first molar crown, and 1.42 mm for the first molar gingival point [19].

Zhou et al. evaluated the correlation between the amount of designed expansion and the efficiency of bodily expansion. The efficiency of expansion decreased from the canine to the first molar [21].

##### Accuracy

In the study by Houle which involved sixty-four patients, the lingual gingival margin at the upper first molar was the area with less accuracy (52.9%). The most reliable area to predict transverse changes in the maxilla was the canine crown with 88.9% of the change achieved. The lower arch presented an overall accuracy of 87.7%, 98.9% at the crown and 76.4% at the gingival margins [19].

In 2020, Morales-Burruezo et al. in a study involving 114 patients found that predictability was 74.8% at the canine, 80.3% at the first premolar, 81% at the second premolar, 79.1% at the first molar, and 65.2% at the second molar [18].

Zhou et al. highlighted that the average expansion efficiencies were 79.75% at the upper canine crown, 76.1% the first premolar crown, 73.27% at the second premolar crown, and 68.31% at the first molar crown [21].

Riede et al. showed that the following differences between simulated and clinical discrepancy were found in the maxillary arch: the largest undercorrection compared to the simulated goals was seen for intermolar width at the gingival margins (2.9 mm) and the largest overcorrection for intercanine width at the gingival margins (3.7 mm) [23].

#### Sagittal movements

In a study of 30 patients, the distalization of upper molars was the most effective movement, with an efficacy of approximately 87%. No statistically significant differences (*p* > 0.05) in terms of accuracy on upper molar distalization (> 1.5 mm) comparing Invisalign® with horizontal beveled gingival attachment and Invisalign® without auxiliaries treatments: 88.4% vs. 86.9%. In the upper arch, the premolars and molars showed the final position more distal than the planned one, whereas in the mandible, the central incisors, the second premolar, and the first molar had the same behavior [11].

Ravera et al. showed that in their 20 participants, the second molar had a distal average movement of 2.52 mm measured on the mesiobuccal cusp and of 2.12 mm measured on the center of the crown, without significant tipping (*P* = 0.056) and vertical movements of the crown (*P* = 0.25). The maxillary central incisor edge was retracted by 2.23 mm (*P* < 0.01) without significant vertical movements (*P* = 0.43) and with a good control of its orientation with respect to the palatal plane (initial value 109.60° ± 6.70°, post-treatment value 106.70° ± 6.66°, *P* < 0.05).^17^ Horizontal movements of all incisors seemed to be accurate, with small (0.20–0.25 mm) or insignificant differences between predicted and achieved amounts [15].

In 2019, Dai et al. in a study with 30 patients, compared achieved and predicted tooth movements of maxillary first molars and central incisors in first premolar extraction cases treated with Invisalign®. First molars achieved greater mesial tipping, mesial translation, and intrusion than predicted. First molars were predicted to tip distally (2.94° ± 3.84°) but actually tipped mesially (2.92° ± 4.62°), with a difference of 5.86° ± 3.5°, and translated mesially 2.26 mm more than predicted [32].

#### Vertical movements

Krieger et al. highlighted that vertical movements were more difficult to reach than transverse or sagittal movements. The parameter overbite displayed the greatest deviations between the predicted and achieved tooth movements (− 0.71 mm) [20].

Gu et al. agreed with this assertion. Moving teeth with aligners is more difficult in the vertical than the sagittal plane, as previously suggested [16].

Extrusion of the maxillary central incisor (56%) was significantly more accurate than intrusion (33%), and intrusion of the mandibular second molar (51%) was significantly more accurate than extrusion (37%) [12].

#### Intrusion

With regard to incisors, the results of the current studies resemble those of others that found movements of anterior teeth to have relatively poor accuracy; thus, significant correction of a deep overbite with Invisalign® appears difficult. Intrusion of incisors was the most inaccurate of all linear movements. The maxillary central incisors had the greatest difference of 1.5 mm (*P*\0.002) [10].

In a study of 120 patients, the authors observed a 1.5-mm median opening of the overbite in the deepbite patients. The primary mechanism responsible for reducing overbite in this group seemed to be the proclination of the mandibular incisors and intrusion of the maxillary incisors. Our results suggested that the mandibular first and second molars were extruded by 0.5 mm on average. Proclination of the mandibular incisors was the main mechanism of bite opening [13].

#### Extrusion

In a study of 120 patients, overbite improved in all patients with pretreatment open bite, with a median deepening of 1.5 mm. Overbite correction in these patients was primarily accomplished by extrusion of the maxillary and mandibular incisors (U1-PP 5 0.9 mm, L1-MP 5 0.8 mm). Extrusion of incisors also appeared to be accurate, since no statistically significant differences were observed. The vertical canine movement seemed to be more predictable in the maxillary arch than in the mandibular arch, although the planned movement for the mandibular arch was greater [13].

#### Rotations

In the study by Simon et al. with 30 patients, premolar derotation showed the lowest accuracy with approximately 40% (SD = 0.3) for rotation of premolars > 10°. No statistically significant differences (*p* > 0.05) could be found between Invisalign® with optimized rotation attachment and Invisalign® without auxiliaries: 37.5% SD 0.3 vs. 42.4% SD 0.3. The results showed that the accuracy was significantly reduced for predicted rotations greater than 15°. Also, the staging had a considerable impact on the treatment efficacy: for rotations with a planned staging > 1.5°/aligner the accuracy was 23% whereas with a staging < 1.5°/aligner the total efficacy was 41.8% [11].

In 2020, Morales-Burruezo et al. showed that virtual planning overestimated the value obtained at the upper right first molar (with a difference close to statistical significance), which corresponded to the real outcome for the upper left first molar. A difference of 2.22 ± 4.37° on the right side and 2.46 ± 3.75° on the left side was identified [18].

All achieved rotations were significantly smaller than those predicted, with the maxillary canines exhibiting the greatest difference of 3.05 (*P*\0.001) [31].

#### Buccolingual movements

##### Torque

In the study by Morales-Burruezo, the results indicated that Invisalign® might not sufficiently produce root torque, especially in the posterior region where the buccolingual inclination is measured [18].

The difference in maxillary central incisor torque found in the current sample was consistent with other studies that observed tipping of incisors rather than bodily movement. In the upper arch, the central and lateral incisors showed a more lingual crown torque than the planned one. Maxillary posterior teeth were positioned more lingual with more facial crown torque than predicted. It is likely that maxillary arch expansion was not fully achieved and the molars tipped rather than moved bodily during the process, both of which could have resulted from flexing of the aligners. The mandibular molars also had more facial crown torque than predicted. This, too, could be the consequence of an inability of the aligners to fully express the torque specified in the virtual treatment plan and may have been compounded by biological limitations such as the proximity of the molar roots to the cortical plate of the mandible [10].

Although previous studies showed that root torque is difficult to control using aligners (especially in the posterior region when compared with the fixed appliances), our results indicated that the buccolingual inclination score was significantly improved after treatment with Invisalign® [28].

In a split-mouth study, the efficacy of orthodontic movements either with or without attachment/power ridge was evaluated. The mean accuracy for upper incisor torque was 42% (SD = 0.2) [11].

No statistically significant differences (*p* > 0.05) in terms of accuracy between planned and clinically obtained movements were found (upper medial incisor torque > 10°), comparing intervention (Invisalign® with horizontal ellipsoid attachments) and control (Invisalign® with power bridges) treatments: 51.5% SD 0.2 vs. 49.1%SD 0.2.

The 11^SnaSnp and 11^Ocl angles showed the highest numeric changes with conventional brackets. The lowest data were reported with aligners [5.13 and 4.60°, respectively]. Conclusions stated that the differences among these techniques were not significant for both angles [33].

##### Proclination

According to Krieger et al., the combination of IPR and incisor protrusion was the main way to correct incisor crowding in 58% of patients [20].

In their retrospective study with 61 patients, Duncan et al. showed that in a more severely crowded dentition, the Invisalign® treatment caused the lower incisor proclination. Lower incisor position and angulation changes were statistically significant in the severe crowding group, but not in the mild and moderate crowding groups [27].

According to the study of Hennessy et al., Invisalign® produced a mean proclination of 3.4 ± 3.2° with respect to fixed appliances which produced 5.3 ± 4.3° of mandibular incisor proclination [30].

Central incisors achieved less retraction and greater lingual crown torque and extrusion than predicted. With regard to upper incisors, in a study on extraction cases with Invisalign®, central incisors tipped more lingually by 5.16° and retracted less by 2.12 mm relative to predicted changes [32].

#### Alignment

##### PAR index

Statistically significant anterior dental crowding improvements (*p* < 0.05) were found both in intervention and control groups in terms of PAR index (22.5 SD 7 to 3.5 SD 3 and 24 SD 6 to 4.5 SD4, respectively) [14].

Both intervention and control groups achieved a statistically significant clinical improvement of PAR index (> 30% of score reduction) (*p* < 0.05). Fixed orthodontic appliance was better than Invisalign® at resolving malocclusion based on PAR index scores (OR 0.33 95%CI 0.13–0.815, *p* = 0.015). A fixed orthodontic appliance was more effective than Invisalign® in reducing the mean percentage of PAR index (*p* = 0.0032). Invisalign® treatment was faster than fixed orthodontic appliance: 13.35 vs 19.08 months, *p* = 0.004 [16].

##### Little index

Statistically significant dental crowding improvements (*p* < 0.05) were also found in terms of the Little irregularity index obtaining a perfect alignment in 92 to 100% of cases in the intervention group and in 88 to 100% of cases in the control group. In Krieger’s work, crowding reduction occurred from 5.39 to 1.57 mm (minimum 0 mm, maximum 4.5 mm) in the upper jaw and from 5.96 to 0.82 mm (minimum 0 mm, maximum 2.50 mm) in the lower jaw. The difference between achieved/predicted tooth movements ranged on average from 0.01 mm (SD ± 0.48) for the lower anterior arch length up to 0.7 mm (SD ± 0.87) for the overbite. All parameters were significantly equivalent except for the overbite (− 1.02, − 0.39) [20].

#### Accuracy

In 2009, Kravitz et al. evaluated the efficacy of anterior tooth movement with Invisalign® and reported an overall mean accuracy of 41%. The most accurate tooth movement was lingual constriction, whereas the least accurate tooth movements were incisor extrusion, followed by a mandibular canine rotation. In 2020, with a prospective clinical study of 38 patients, the mean accuracy of Invisalign® for all tooth movements was 50%. The highest overall accuracy was achieved with a buccal-lingual crown tip (56%), whereas the lowest overall accuracy occurred with rotation (46%) [34].

#### Aligners’ change

In an uncontrolled clinical trial of 37 participants, Drake et al. showed that a large part of the movement occurs in the first week. No significant difference over an 8-week time period was found in the amount of OTM (orthodontic tooth movement) between those who wore the same aligner for 2 weeks compared to those who changed to a new duplicate aligner after one week. The role of uncontrolled tipping and loss of anchorage complicated the progression of programmed aligners [26].

In their study with 30 participants, Chisari et al. revealed similar findings. Most tooth movement occurred in the first week of the 2-week wear cycle. Although it was not statistically significant (*P* = 0.06), participants with a smaller goal had a higher mean percentage of goal achieved, 62%, compared with 54% for those with a planned movement of 0.50 mm. The combined data indicate that despite having aligners programmed to move 1 central incisor 1 mm labially (0.25 mm per aligner), on average only 57% of that movement was achieved. It has been postulated that a greater percentage of tooth movement would occur if the prescription in each aligner was decreased from 0.5 to 0.25 mm. As mentioned earlier, the magnitude and direction of force placed on teeth during OTM, in addition to the length of time these forces are in place, can play critical roles in how teeth move [29].

## Discussion

Over the last years, the results of studies have focused on the achievement of the Invisalign® system in terms of mm or degrees obtained in planned movement. ClinCheck® software is not only evaluated in the context of dental movement but also in reference to the system of forces behind it. Respecting the protocols and precise staging, dental movement is allocated in the different phases of treatment due to the use of an algorithm and through a system of forces that allows the expected movements. In the literature, although many are the systematic reviews that have evaluated the accuracy of movements with Invisalign®, updated studies in line with the progress of this technique have to be taken into account. Evaluating the efficacy of anterior tooth movement with Invisalign®, Kravitz et al. (2009) detected an overall mean accuracy of 41%. According to a prospective study about all tooth movements carried out in 2020, the accuracy increased up to 50%. In both studies, true incisor extrusion resulted to be the least accurate tooth movement, followed by the rotation of the mandibular canine, whereas lingual constriction was reported to be the most accurate movement. The authors suggested that combining extrusion with lingual crown tip (relative extrusion) allows more predictable movements [17, 26, 29, 34, 35]. Since the buccal and lingual aspects of the crown provide the largest surface area to push, it is a logical consequence that the most predictable results are due to the bucco-lingual movements. The more flexible SmartForce aligner material together with the power ridges has brought an improved accuracy in the incisor buccal crown tip [29]. Notwithstanding this, problems could arise for the second molars due to the poor aligner grip around the shorter terminal crown but also to the decreased forces on the terminal tooth within the aligner. As it concerns the rotation of rounded teeth, problems were not completely solved even though optimized attachments were used for rotational movements greater than 5°. Furthermore, the accuracy of mesial rotation (52%) was significantly better than distal rotation (37%) [29]. Similar results were observed by Simon et al. and Charalampakis et al. [10, 11]. A further evaluation of the efficacy of premolar derotation was performed taking into consideration both the amount of tooth movement and the amount of staging planned. The results related to predicted rotations greater than 15° as well as for rotations with a planned staging > 1.5°/aligner showed that the accuracy was significantly reduced [11]. As it concerns the movement accuracy, the literature shows that it was particularly low: exactly up to 28% for mesial rotation of the mandibular first molar, a little more up to 37% for distal rotation of the maxillary canine, and 35% for the intrusion of the mandibular incisors. The last result was the same as reported by Grunheid et al. The lack of posterior anchorage may be one of the explanations for the lower accuracy of mandibular incisor intrusion. In contrast, the accuracy of the second molar intrusion (51%) was relatively high. As a consequence, it can be confirmed that Invisalign® is more effective in bite closure, rather than bite opening [24, 29, 35]. ClinCheck® prediction of expansion involves more bodily movement of the teeth than can be seen clinically. Furthermore, more dental tipping was observed at the end of the treatment so it follows that a careful planning with overcorrection and other auxiliary methods of expansion which may help reduce the rate of midcourse corrections and refinements, especially in the posterior region of the maxilla is suggested [17, 18, 36]. Duncan et al. showed that crowding in the maxillary arch is corrected using IPR in most of the cases, whereas crowding in the mandible is corrected with IPR in 30% of the cases, IPR with protrusion of the incisors in 40% of cases, and sole protrusion of the incisors in 18%. Post-Invisalign® treatment showed that 58% of the patients had had some increase in mandibular arch length [27]. A study about the recovery of space in mild crowding cases conducted in 2016 by Hennessy et al. showed that fixed appliances produced 5.3 ± 4.3° of mandibular incisor proclination whereas Invisalign® proclined the mandibular incisors by 3.4 ± 3.2°. No statistically significant difference was detected between the two groups [30]. It has to be considered that when treating crowded dentitions, in order to negate or minimize lower incisor proclination, buccal expansion(if indicated) and IPR are important clinical tools to be used [27]. In conclusion, two research studies revealed that over an 8-week time period, no significant difference was found in the amount of OTM (orthodontic tooth movement) between those who wore the same aligner for 2 weeks if compared to those who changed to a new duplicate aligner after 1 week. However, the reduction in the amount of OTM detected during the second week was not due to material fatigue [26, 29].

### Limitations

It was not possible to carry out the meta-analysis due to the great heterogeneity of the parameters used for the same outcome for the selected studies. Therefore, for some of the outcomes (PAR Index, Little Index, Aligners Change), there are few studies which analyzed each of them.

## Conclusions

Although this review included a considerable number of studies, no clear clinical recommendations can be made, based on solid scientific evidence, apart from non-extraction treatment of mild to moderate malocclusions in non-growing patients. There is still a shortage of high-quality evidence concerning the treatment modality. The introduction of SmartForce and SmartTrack material has improved the efficacy of the treatment, but how studies’ findings are reported is unclear.

Clinicians should consider the following indications for aligner treatment:

-Expansion of the upper arch occurs through more coronal tipping than bodily movement. The predictability of coronal expansion decreases moving towards the posterior sector.
-The predictability in the deepbite correction is reduced, thus requiring greater attention in the planning of the ClinCheck® software. It occurs mainly through the proclination of the lower incisors (relative intrusion).
-Differently, in the treatment of open bite, the resolution occurs through a combination of extrusion of the incisors and lingual crown tip (relative extrusion).
-In cases of crowding, the correct management involves the combination of expansion and IPR to reduce the incisal proclination.
-In sagittal movements, it is advisable not to go beyond the 2–3 mm distalization of the molars.
-At the end of the treatment, the incisal position is almost always more occlusal than expected, the rotations of the premolars and the incisal torque are not completely resolved.

The authors want to emphasize to that almost all the published scientific literature presents biases since there is not any knowledge about the clinical level of those who use the software to plan the treatment. Moreover, the latest features of the system and the latest digital protocols are not taken into account. All things considered, it is evident that more high-quality research of prospective design focused on force system that leads to orthodontic movement in each phase of staging needs to be carried out in the future. Moreover, the major problem is that a digital biomechanics system is compared to traditional biomechanics and that the final results of the software are measured considering it as a final position to be reached and not as a system of forces to be applied.

Until the evaluation trend of the Invisalign® system changes, the scientific literature will be limited to the evaluation of the technique itself.

## Data Availability

The dataset(s) supporting the conclusions of this article is(are) included within the article (and its additional file(s).
